# CUSUM learning curves: what they can and can’t tell us

**DOI:** 10.1007/s00464-023-10252-1

**Published:** 2023-07-17

**Authors:** Peng-Lin Lin, Feibi Zheng, Minkyung Shin, Xi Liu, Daniel Oh, Daniel D’Attilio

**Affiliations:** Intuitive Surgical, 1020 Kifer Road, Sunnyvale, CA 94086-5304 USA

**Keywords:** Learning curve, CUSUM, Console time, Robotic-assisted surgery

## Abstract

**Introduction:**

There has been increased interest in assessing the surgeon learning curve for new skill acquisition. While there is no consensus around the best methodology, one of the most frequently used learning curve assessments in the surgical literature is the cumulative sum curve (CUSUM) of operative time. To demonstrate the limitations of this methodology, we assessed the CUSUM of console time across cohorts of surgeons with differing case acquisition rates while varying the total number of cases used to calculate the CUSUM.

**Methods:**

We compared the CUSUM curves of the average console times of surgeons who completed their first 20 robotic-assisted (RAS) cases in 13, 26, 39, and 52 weeks, respectively, for their first 50 and 100 cases, respectively. This analysis was performed for prostatectomy (1094 surgeons), malignant hysterectomy (737 surgeons), and inguinal hernia (1486 surgeons).

**Results:**

In all procedures, the CUSUM curve of the cohort of surgeons who completed their first 20 procedures in 13 weeks demonstrated a lower slope than cohorts of surgeons with slower case acquisition rates. The case number at which the peak of the CUSUM curve occurs uniformly increases when the total number of cases used in generation of the CUSUM chart changes from 50 to 100 cases.

**Conclusion:**

The CUSUM analyses of these three procedures suggests that surgeons with fast initial case acquisition rates have less variability in their operative times over the course of their learning curve. The peak of the CUSUM curve, which is often used in surgical learning curve literature to denote “proficiency” is predictably influenced by the total number of procedures evaluated, suggesting that defining the peak as the point at which a surgeon has overcome the learning curve is subject to routine bias. The CUSUM peak, by itself, is an insufficient measure of “conquering the learning curve.”

The concept of learning curves is commonly employed to model performance variables as a function of time or experience. First introduced in the aircraft industry by Wright in 1936 [1], learning curves were developed based on the observation that man-hour requirements decreased uniformly as the number of individual units produced doubled. In other words, workers’ performance improved with time and experience.

This idea has since been applied to healthcare and surgery, with the surgical learning curve representing the relationship between a surgeon’s experience and the outcomes of a surgical procedure [2]. As novel minimally invasive surgical techniques and technologies have proliferated, interest in evaluating surgeons’ learning curve progression has grown. Although numerous studies have measured surgical learning curves for various procedures, inconsistencies in statistical methods and outcome measures exist [3, 4]. Generally, surgical learning curve methodologies can be classified into four categories: graphical inspection, split-group, cumulative sum (CUSUM), and regression. These methods can be used in combination, and each possesses distinct advantages and limitations [5, 6].

Of the four methods, CUSUM is among the most frequently employed. H. Page first introduced the CUSUM technique in 1954 as a quality control tool to detect small changes in the process mean over time [7]. The technique has since been further developed and refined, finding application across various fields, including surgical learning curve assessment [8]. CUSUM involves the continuous summation of differences between the outcome of interest for each case and the target value. These outcomes may include operative times (OR time) or other continuous procedural or patient outcome variables. In the literature, OR time and average OR time are the most frequently used for outcome of interest and target variable, respectively. [9, 10]. One advantage of CUSUM methodology is its visual appeal: it frequently produces an inverse parabolic curve with a clear peak. Consequently, CUSUM has become an increasingly popular method for establishing when surgeons have “conquered the learning curve.”

However, learning curves can be impacted by many factors not accounted for in a traditional CUSUM calculation [11]. First, there is considerable heterogeneity in the standard definition of the optimal statistical method, as the choice of outcome measures and target values can influence results. Second, many studies lack representativeness, as they typically examine the learning curves of one or a few surgeons [9]. The learning curve can vary significantly among individual surgeons due to factors such as prior training in other surgical modalities, operative experience in other procedures, and simulation or coaching, which can potentially shorten the learning curve [12, 13]. Additionally, the frequency of performing a procedure (case cadence) may impact learning curves, an aspect not thoroughly examined in the literature. Lastly, patient complexity, case difficulty, and team dynamics are not typically incorporated into a CUSUM model.

This study aims to analyze surgeons’ learning curves with varying case acquisition rates and examine the mathematical properties of CUSUM. We assessed CUSUM console time across cohorts of surgeons with different case acquisition rates, while varying the total number of cases used to calculate the CUSUM. In doing so, we demonstrate the limitations of this methodology.

## Materials and methods

### CUSUM

The cumulative sum (CUSUM) method, which uses the average console times across the procedure index as the target value, was employed. We inspected console times for the first $i$ procedures

$${S}_{i}={\sum }_{j=1}^{i}\left({Y}_{j}-\overline{Y }\right),$$

where ${S}_{i}$ represents the CUSUM score of the* ith* procedure, ${Y}_{j}$ represents the average console time of the *jth* procedure for the surgeons, and $\overline{Y }$ represents the average of ${Y}_{j}$ in each cohort. The procedure index at the peak of the CUSUM learning curve is considered the number of cases required for surgeons to “conquer the learning curve.”

### Console time

Anonymized surgical console times were extracted from the da Vinci system’s log data, provided by Intuitive Surgical, for three procedures: inguinal hernia, prostatectomy, and malignant hysterectomy. Considering that this is an observational study using deidentified system log data with no possibility of identification of patients, institutional review board was not required.

All surgeons whose data were included in the study had completed at least 100 cases for each procedure. To our knowledge, this is the first attempt to group the surgeons based on the case acquisition rate. We initially examined the frequency of inguinal hernia, prostatectomy, and malignant hysterectomy surgeries using data from the da Vinci system’s logs. We found that over 10% of surgeons were present in each quarter of the first year (13, 26, 39, and 52 weeks), but the percentage dropped to less than 10% from the fifth quarter (Table 1). Additionally, most surgeons completed their first 20 cases within one year (79.9% for inguinal hernia, 71.8% for prostatectomy, and 67.5% for malignant hysterectomy). Consequently, we decided to categorize surgeons into four groups based on the time taken to complete their first 20 robotic-assisted surgeries (RAS): 13 (1.5 cases/week), 26 (0.76 cases/week), 39 (0.51 cases/week), and 52 (0.38 cases/week) weeks. Surgeons who could not be categorized into these four groups were excluded from the analysis (e.g., surgeons who had not finished 100 cases of the procedure of interest or took more than 52 weeks to finish their first 20 cases).

**Table 1:** The percentage of the surgeons completed their first 20 cases by quarter

	Time to first 20 cases				
	1 quarter (%)	2 quarters (%)	3 quarters (%)	4 quarters (%)	5 + quarters (%)
Inguinal hernia	22.2	31.4	15.6	10.7	All < 10
Prostatectomy	14.9	26.4	19.5	11.0	All < 10
Malignant hysterectomy	11.8	25.4	18.2	12.1	All < 10

### Statistical analysis

The average console times for each procedure index in each group were calculated, and line charts were used to inspect the changes in average console times over the procedure index for the first 50 and 100 cases. CUSUM scores for each procedure index were calculated based on the average console times. CUSUM plots were then created for the first 50 cases and for the first 100 cases separately. The peak of the CUSUM curve was defined as the maximum value on the curve. We performed the Kolmogorov–Smirnov test to compare the difference of CUSUM curves across cohorts in each procedure type.

## Results

The number of surgeons in each group is presented in Table 2. A total of 1486 surgeons were included in the analysis for inguinal hernia, 1094 for prostatectomy, and 737 for malignant hysterectomy.

**Table 2:** The number of surgeons in each cohort

	Time to first 20 cases				Total
	13 weeks	26 weeks	39 weeks	52 weeks	
Inguinal hernia	495	589	277	125	1486
Prostatectomy	288	430	254	122	1094
Malignant hysterectomy	167	291	173	106	737

### Inguinal hernia

**Fig. 1:**
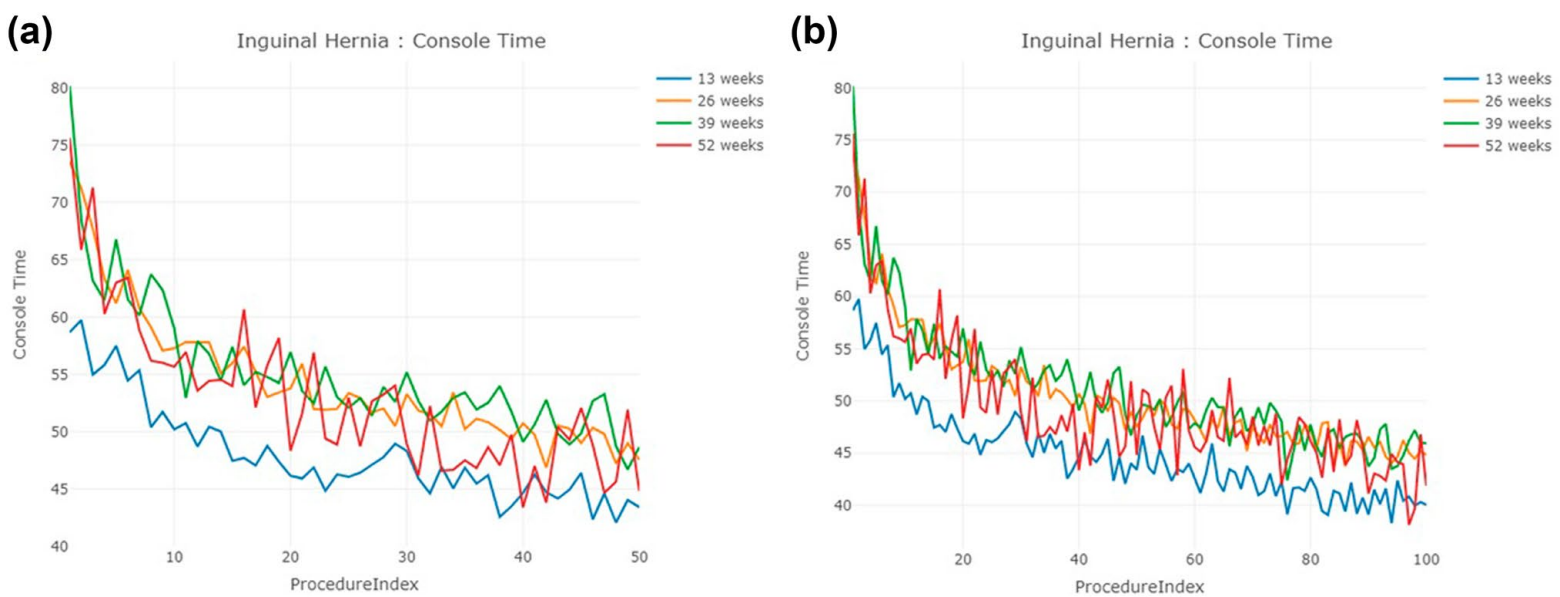
Console time trends of inguinal hernia for the first **a** 50 cases, **b** 100 cases

A total of 495, 589, 277, and 125 surgeons completed their first 20 cases in 13, 26, 39, and 52 weeks, respectively. The console time trends of their first 50 and 100 cases are presented in Fig. 1a and b. All trends show a similar learning curve shape, characterized by initial rapid improvement followed by a leveling off as surgeons approach a plateau. Notably, compared to the other three groups, surgeons who completed their first 20 cases within 13 weeks had the shortest console time from start to finish. The 13-week group reached its average console time for the first 50 cases (47.9 min) at case number 16. In contrast, it took 43, 50, and 32 cases for the other three groups to first reach 47.9 min. Furthermore, even with a relatively large number of surgeons in the 13-week cohort, the standard deviation was the smallest among the four groups (Table 3).

**Table 3:** Average console times in minutes across the procedure index

	Time to first 20 cases				*p*-value
	13 weeks	26 weeks	39 weeks	52 weeks	
*Inguinal hernia*					
First 50 cases (mean, sd)	47.9 (4.2)	54.4 (5.8)	55.1 (5.9)	52.9 (6.8)	< 0.0001
First 100 cases (mean, sd)	44.9 (4.6)	50.6 (5.7)	51.2 (5.9)	49.4 (6.3)	< 0.0001
*Prostatectomy*					
First 50 cases (mean, sd)	124.5 (3.7)	139.0 (4.4)	144.9 (7.9)	148.1 (8.1)	< 0.0001
First 100 cases (mean, sd)	121.9 (4.4)	135.2 (5.1)	138.8 (8.7)	142.4 (8.7)	< 0.0001
*Malignant hysterectomy*					
First 50 cases (mean, sd)	72.6 (3.4)	90.5 (3.6)	90.6 (6.2)	94.9 (7.6)	< 0.0001
First 100 cases (mean, sd)	70.8 (3.8)	87.2 (4.7)	87.0 (6.4)	90.1 (8.3)	< 0.0001

**Fig. 2:**
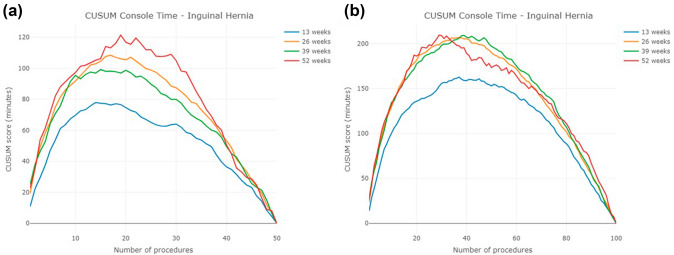
CUSUM learning curve of inguinal hernia for the first **a** 50 cases, **b** 100 cases

After transforming the console time into CUSUM scores, the CUSUM learning curve of the first 50 and 100 cases of inguinal hernia is shown in Fig. 2a and b. The CUSUM curve of the cohort of surgeons who completed their first 20 procedures in 13 weeks demonstrated a lower slope than cohorts of surgeons with slower case acquisition rates. The results are statistically significant (Table 4). However, the CUSUM curves of the other three cohorts are similar to each other. When examining the first 50 cases, surgeons reach their CUSUM peak at their 14th, 17th, 20th, and 19th cases in the 13, 26, 39, and 52-week groups, respectively. However, when examining the first 100 cases, the case number at which the CUSUM peak occurred increased to 37, 35, 39, and 29 cases for the four cohorts, respectively (Table 5). In other words, the CUSUM analysis provided two different answers for the number of cases needed to “conquer the learning curve” among the same surgeons, simply by changing the number of procedures evaluated.

**Table 4:** Comparison of CUSUM curves using Kolmogorov–Smirnov test

	13 weeks vs 26 weeks	13 weeks vs 39 weeks	13 weeks vs 52 weeks	26 weeks vs 39 weeks	26 weeks vs 52 weeks	39 weeks vs 52 weeks
*50 cases*						
Inguinal hernia	< 0.0001	0.0002	< 0.0001	0.1786	0.9667	0.1124
Prostatectomy	< 0.0001	< 0.0001	< 0.0001	< 0.0001	< 0.0001	0.0006
Malignant hysterectomy	< 0.0001	< 0.0001	< 0.0001	0.0006	< 0.0001	< 0.0001
*100 cases*						
Inguinal hernia	< 0.0001	< 0.0001	< 0.0001	0.9996	0.6994	0.5806
Prostatectomy	< 0.0001	< 0.0001	< 0.0001	< 0.0001	< 0.0001	0.0002
Malignant hysterectomy	< 0.0001	< 0.0001	< 0.0001	< 0.0001	< 0.0001	< 0.0001

**Table 5:** Number of cases to conquer the surgical learning curve using CUSUM

	Time to first 20 cases			
	13 weeks	26 weeks	39 weeks	52 weeks
*Inguinal hernia*				
First 50 cases	14	17	20	19
First 100 cases	37	35	39	29
*Prostatectomy*				
First 50 cases	17	19	24	21
First 100 cases	49	42	35	46
*Malignant hysterectomy*				
First 50 cases	19	29	21	27
First 100 cases	47	42	39	43

### Prostatectomy

**Fig. 3:**
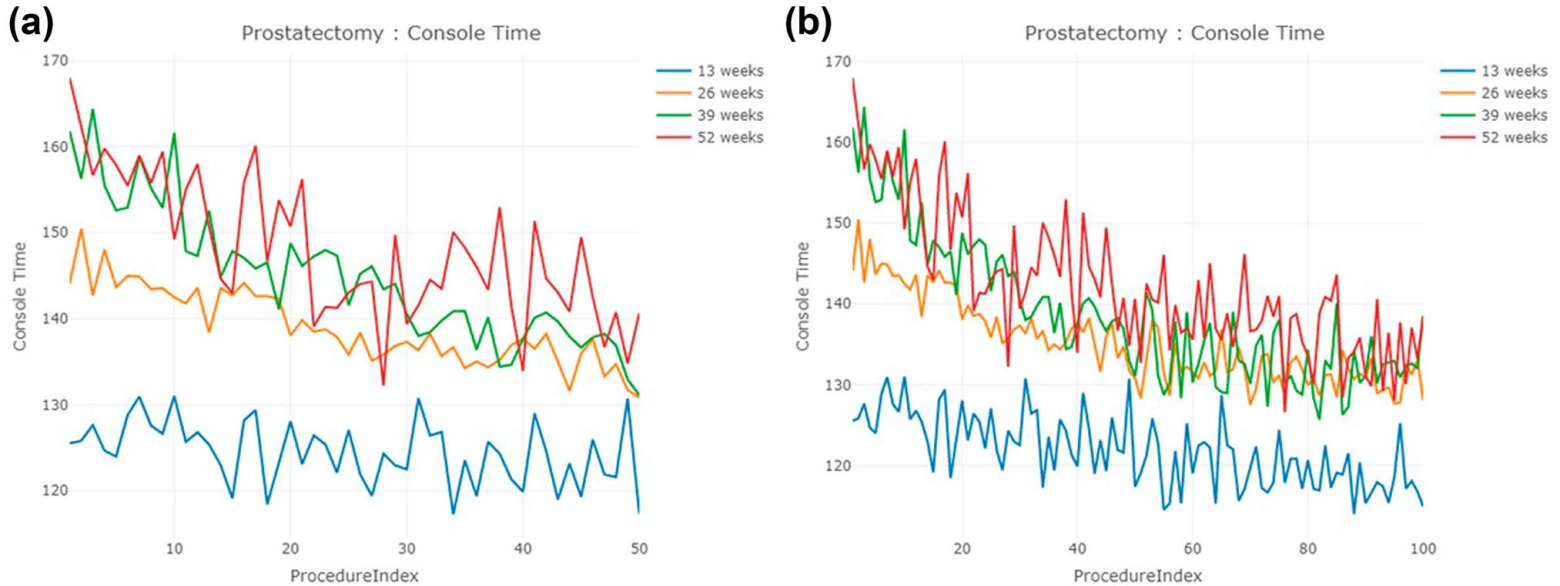
Console time trends of prostatectomy for the first **a** 50 cases, **b** 100 cases

The number of surgeons in each cohort is 288, 430, 254, and 122 in 13-, 26-, 39-, and 52-week cohort, respectively (Table 2). Figure 3a and b present the console time trends for the first 50 and 100 cases in each group. The console time trend of the 13-week cohort appears to be stable, as they reached an average console time of 124.5 min for their first 50 cases by their 5th case. In contrast, the other three cohorts failed to achieve an average console time of 124.5 min across their first 100 cases. Similar to inguinal hernia, surgeons who completed their first 20 cases in 13 weeks had the shortest average console time compared to the other groups. Additionally, the average console times of the 26-week group were shorter than those of the 39-week group, followed by the 52-week group. The standard deviation of console times followed the same trend, with the 13-week cohort demonstrating the smallest standard deviation, followed by the 26-week, 39-week, and then 52-week cohorts (Table 3).

**Fig. 4:**
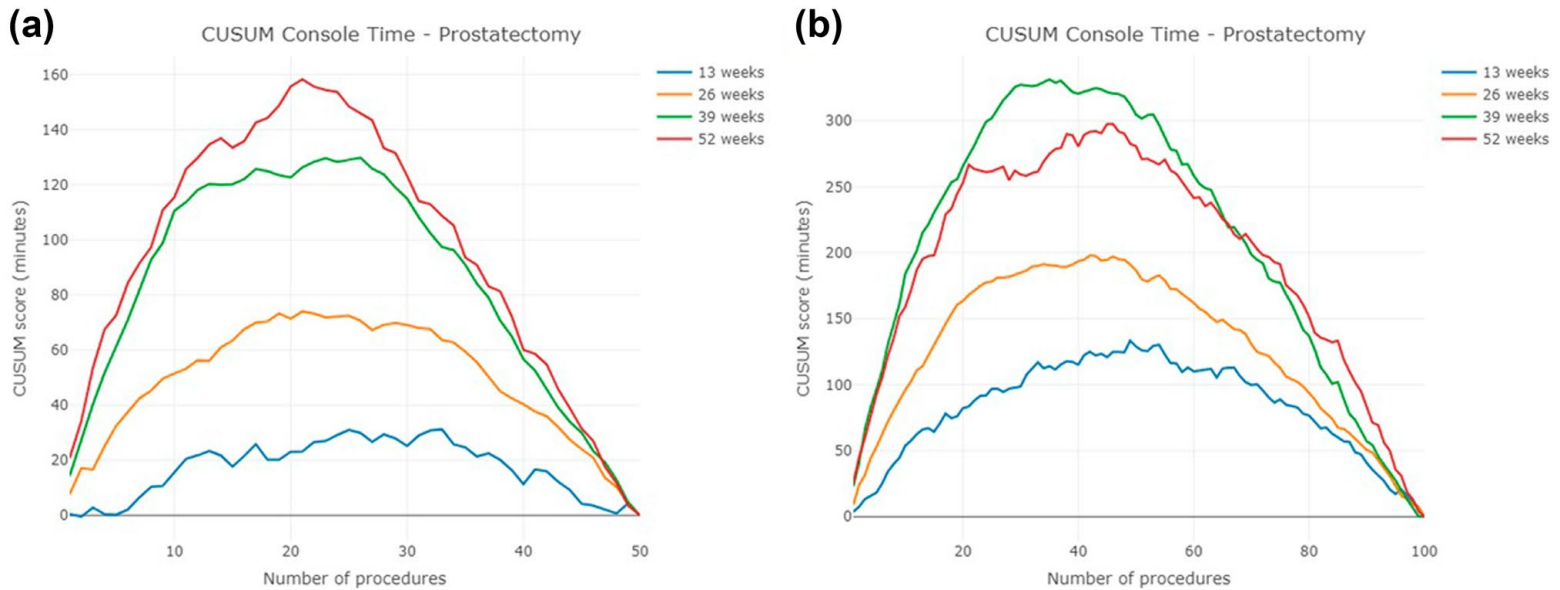
CUSUM learning curve of prostatectomy for the first **a** 50 cases, **b** 100 cases

The CUSUM learning curves are presented in Fig. 4a and b. Again, the 13-week cohort exhibited the smallest slope and the most stable CUSUM curve in both 50 and 100 case analyses. Interestingly, the CUSUM curve of the 39-week cohort appears to be higher than that of the 52-week cohort until case number 69 is reached. When analyzing the first 50 cases, the number of cases of the inflection point of CUSUM are 17, 19, 24, and 21 in the four cohorts. However, the number of cases increases to 49, 42, 35, and 46 when studying the first 100 cases (Table 5).

### Malignant hysterectomy

**Fig. 5:**
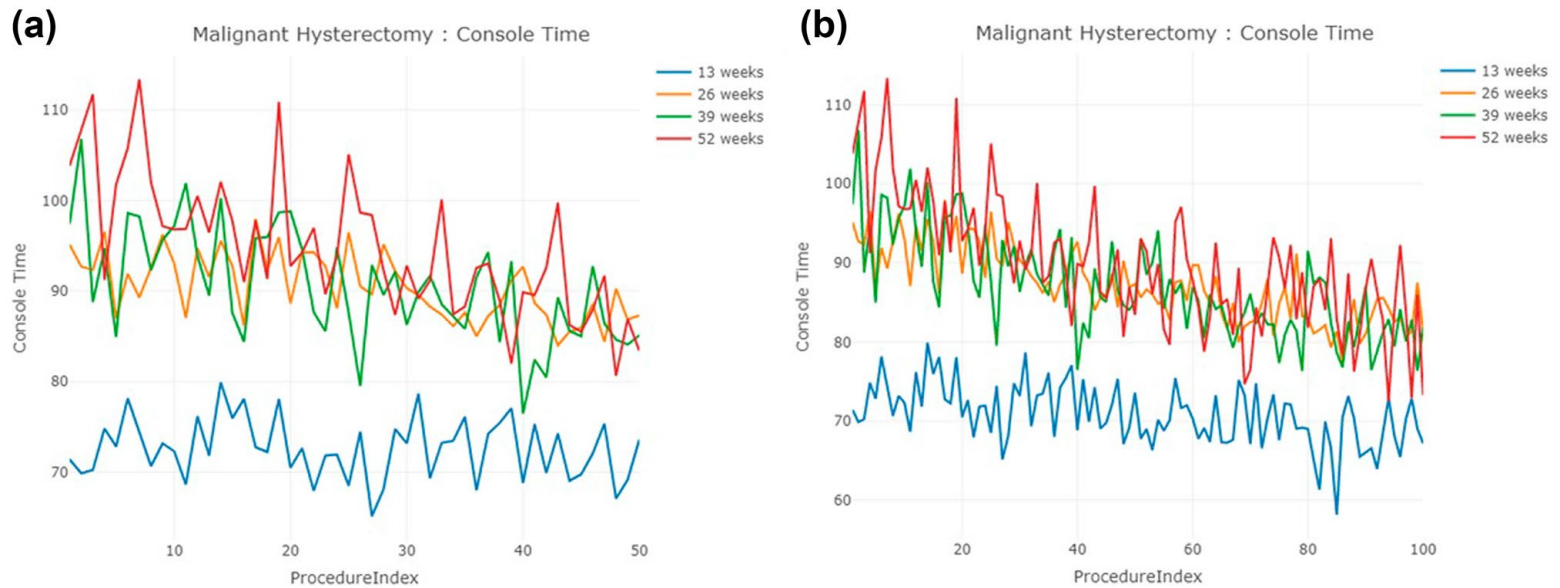
Console time trends of malignant hysterectomy for the first **a** 50 cases, **b** 100 cases

The number of surgeons in each cohort is 167, 291, 173, and 106, respectively (Table 1). The 13-week cohort of surgeons exhibited the shortest console time and the smallest standard deviations (Table 2), with the console time trend also significantly shorter than the other three cohorts. However, the trends of the other three cohorts are difficult to distinguish due to fluctuations in the line chart (Fig. 5a and b). Like the results observed in prostatectomy, the trend for the 13-week cohort in this study was consistent, with an average console time of 72.6 min achieved at their first case. In contrast, the 26-, 39-, and 52-week cohorts failed to achieve an average console time of 72.6 min across their first 100 cases.

**Fig. 6:**
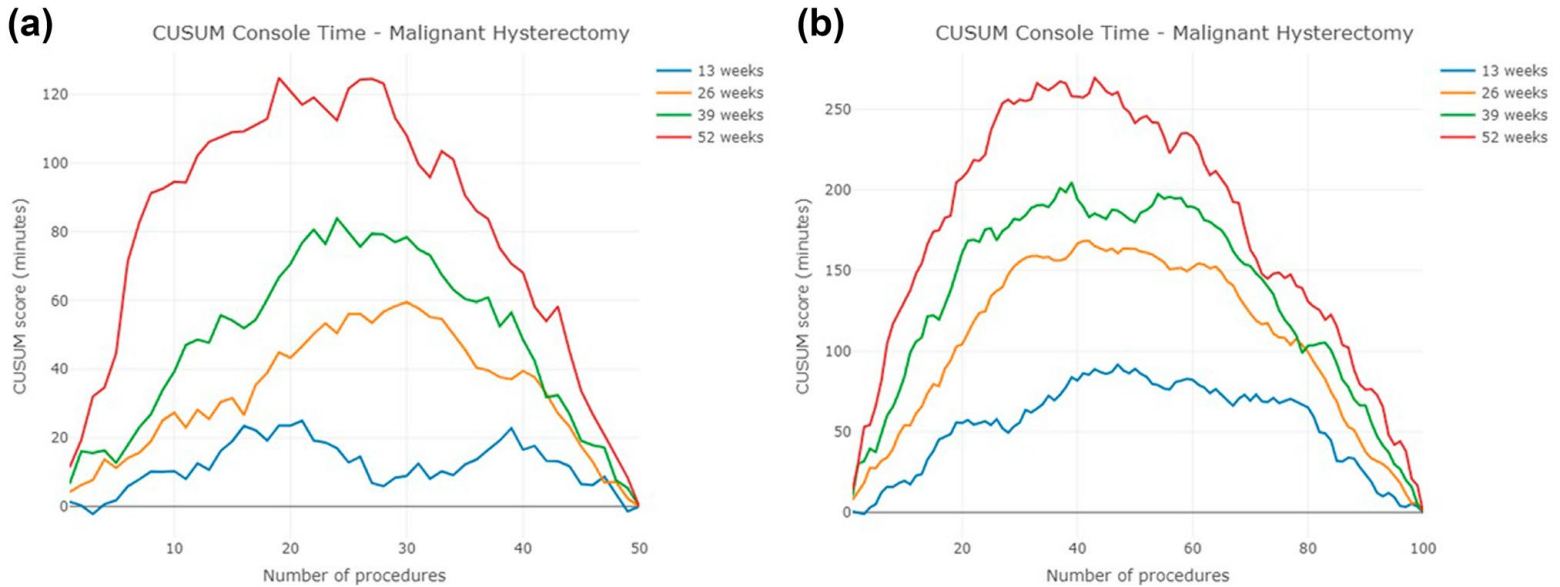
CUSUM learning curve of malignant hysterectomy for the first **a** 50 cases, **b** 100 cases

When transforming the console time trends into CUSUM learning curves, it becomes evident that the height of the CUSUM learning curve is generally lower in the 13-week cohort, followed by the 26, 39, and 52-week cohorts (Fig. 6). Again, the CUSUM learning curves present two sets of answers regarding the number of cases required for surgeons to conquer the learning curve (Table 3).

## Discussion

This study examines the learning curve of robotic-assisted surgeries in inguinal hernia, prostatectomy, and malignant hysterectomy. We illustrate the relationship between the learning curve and the case acquisition rate, while also revealing the limitations of the CUSUM technique. Our findings highlight several important aspects of the CUSUM learning curve and its implications in surgical practice.

Our results indicate that surgeons who completed their first 20 cases within a shorter timeframe (13 weeks) demonstrated shorter console times and smaller standard deviations. This finding is consistent with the notion that more frequent practice and exposure to surgical procedures during the early stages of training can lead to a faster improvement in surgical skills. The CUSUM analyses of these three procedures suggest that surgeons with fast initial case acquisition rates have less variability in their console times over the course of their learning curve. The original console time trends support the same conclusions. However, there are some minor differences across the three procedures concerning the relationship between the learning curve and the case acquisition rate.

In the case of inguinal hernia, the cohort that completed the first 20 cases in 13 weeks demonstrated shorter console times and smaller standard deviations than the other three cohorts, but there is no significant difference regarding the CUSUM learning curve across the other three cohorts. In contrast, both in prostatectomy and malignant hysterectomy, the four cohorts exhibit pairwise significantly different learning curves. To our knowledge, this is the first learning curve study to categorize surgeons based on the case acquisition rate. This difference suggests that a standardized method to group surgeons based on the case acquisition rate is needed.

In the literature, the reported case numbers for conquering the learning curve using operative time for each procedure are highly inconsistent. In inguinal hernia, the reported number of cases until the CUSUM peak is reached ranges from 43 to 138 for RAS and 30 for LAP surgery [14–16]. The discrepancy for prostatectomy is even larger, ranging from 40 to 1000 cases (RAS: 40–100; LAP: 200–750; Open: 250–1000) [11, 17, 18]. For minimal-invasive hysterectomy, the range is from 10 to 36 [19–22]. As most learning curve studies are retrospective, they are likely subject to confounding factors such as patient comorbidity, selection, the level of case complexity, and surgeon’s baseline skill and previous experience [12]. These factors could also contribute to the observed differences in learning curve progression among the different cohorts.

Interestingly, our study introduces two additional potential explanations for this discrepancy: (1) the learning curve is highly influenced by the initial case acquisition rate; and (2) our analyses reveal the fundamental flaws in using self-referring statistics as a target value for the CUSUM method. The CUSUM peak is predictably affected by the total number of procedures included in the analysis. In the literature, the peak is used to decide when surgeons conquer the learning curve. However, we observed that the case number at which the peak of the CUSUM curve occurs uniformly increases when the total number of cases used in generating the CUSUM chart changes from 50 to 100 cases. This is due to the mathematical properties of CUSUM and the way we chose the target values.

Originally, the CUSUM was designed to use the external standard value as the target value [7, 23–25]. However, in the literature, the most common target value for operative time is the sample mean [8]. As long as the console time gradually decreases as the case number increases, the CUSUM plot will follow a quadratic form. That is, the shape of the CUSUM curve will usually go up and then go down until the CUSUM score reaches zero. Since the average is calculated from the data itself, the target value becomes a self-reference value, and the case number of the CUSUM peak is nothing more than the case number when the console time reaches the average console time. Furthermore, because of the quadratic shape tendency of CUSUM, the peak always happens around the middle of the curve. In other words, the more procedures we observe, the longer it takes for the surgeon to reach the CUSUM peak. Thus, using the “peak” of CUSUM to determine whether surgeons reach proficiency is subject to routine bias.

Finally, it should be noted the CUSUM plot can often be misleading and thus misinterpreted. Our results of the first 100 cases of prostatectomy demonstrate that the CUSUM curve for the 39-week cohort was higher than that of the 52-week cohort until case number 69. This observation shows that in the early phase of learning, the 39-week cohort has a higher accumulated variation than the 52-week cohort. However, a higher variation does not necessarily reflect the learning curve accurately. If we inspect the console time trends of the two cohorts, we can identify that the 39-week cohort performed better than the 52-week cohort. The average console time of the 39-week cohort is shorter than that of the 52-week cohort (39-week: 138.8 min; 52-week: 142.4 min) with the same standard deviation (8.7 min). Thus, the CUSUM curve may not always accurately represent the true learning progression of surgeons, especially when comparing different cohorts with varying case acquisition rates.

There are some limitations to this study. First, we only use console time to study the robotic surgical learning curve. Since system log data does not record any clinical information about the patient, we could neither track the performance of other clinical outcomes through the learning curves nor could we adjust for patient baseline characteristics. Second, a surgeon’s previous experience with open or laparoscopic surgeries may have been a significant factor for the robotic learning curve [9]. However, there is no such information regarding the surgeons’ previous experiences with other surgical modalities in system data. Because the surgeons who completed their first 20 robotic cases within 13 weeks have the shortest console time from their very first case, we suspect that the surgeons in the fast case acquisition cohort may have better previous experience in open or laparoscopic surgeries than the other groups or are higher volume surgeons when considering all modalities.

In conclusion, it is crucial to recognize the limitations of the CUSUM method in studying surgical learning curves. The peak of the CUSUM curve, frequently employed in surgical learning curve studies to indicate “proficiency,” is predictably affected by the total number of procedures analyzed. This implies that using the peak as the point where a surgeon has mastered the learning curve may be prone to regular bias. The CUSUM peak alone is an inadequate indicator of successfully “overcoming the learning curve.” Future research should focus on developing more robust and standardized methods to assess surgical learning curves, considering the complexity of surgeries, patients’ baseline characteristics, and other confounding factors that may impact the learning progression of surgeons. Our findings suggest that case cadence has an impact on console time variability over the entire learning curve. This finding should be considered when designing training programs for new techniques and new technologies.
